# Laisk measurements in the nonsteady state: Tests in plants exposed to warming and variable CO_2_ concentrations

**DOI:** 10.1093/plphys/kiad305

**Published:** 2023-05-26

**Authors:** Stephanie C Schmiege, Thomas D Sharkey, Berkley Walker, Julia Hammer, Danielle A Way

**Affiliations:** Plant Resilience Institute, Michigan State University, East Lansing, MI 48824, USA; Department of Biology, Western University, London, Ontario N6A 5B7, Canada; Plant Resilience Institute, Michigan State University, East Lansing, MI 48824, USA; MSU-DOE Plant Research Laboratory, Michigan State University, East Lansing, MI 48824, USA; Department of Biochemistry and Molecular Biology, Michigan State University, East Lansing, MI 48824, USA; MSU-DOE Plant Research Laboratory, Michigan State University, East Lansing, MI 48824, USA; Department of Plant Biology, Michigan State University, East Lansing, MI 48824, USA; Department of Biology, Western University, London, Ontario N6A 5B7, Canada; Department of Biology, Western University, London, Ontario N6A 5B7, Canada; Research School of Biology, The Australian National University, Acton, Australian Capital Territory 2601, Australia; Nicholas School of the Environment, Duke University, Durham, NC 27710, USA; Environmental & Climate Sciences Department, Brookhaven National Laboratory, Upton, NY 11973, USA

## Abstract

Light respiration (*R*_L_) is an important component of plant carbon balance and a key parameter in photosynthesis models. *R*_L_ is often measured using the Laisk method, a gas exchange technique that is traditionally employed under steady-state conditions. However, a nonsteady-state dynamic assimilation technique (DAT) may allow for more rapid Laisk measurements. In 2 studies, we examined the efficacy of DAT for estimating *R*_L_ and the parameter *C*_i_* (the intercellular CO_2_ concentration where Rubisco's oxygenation velocity is twice its carboxylation velocity), which is also derived from the Laisk technique. In the first study, we compared DAT and steady-state *R*_L_ and *C*_i_* estimates in paper birch (*Betula papyrifera*) growing under control and elevated temperature and CO_2_ concentrations. In the second, we compared DAT-estimated *R*_L_ and *C*_i_* in hybrid poplar (*Populus nigra* L. × *P. maximowiczii* A. Henry “NM6”) exposed to high or low CO_2_ concentration pre-treatments. The DAT and steady-state methods provided similar *R*_L_ estimates in *B. papyrifera*, and we found little acclimation of *R*_L_ to temperature or CO_2_; however, *C*_i_* was higher when measured with DAT compared to steady-state methods. These *C*_i_* differences were amplified by the high or low CO_2_ pre-treatments. We propose that changes in the export of glycine from photorespiration may explain these apparent differences in *C*_i_*.

## Teaser

Nonsteady-state Laisk measurements provide similar estimates of light respiration to steady-state methods but can be completed 4 times faster.

## Introduction

Respiration in the light (*R*_L_) and Γ* (the CO_2_ compensation point in the absence of *R*_L_) are 2 parameters critical to biochemical models of photosynthesis (Farquhar et al. 1980; von Caemmerer 2000), and their accurate estimation is required to understand current and future carbon cycling across scales (e.g. Wohlfahrt et al. 2005; Rogers et al. 2017). Despite the importance of *R*_L_ and Γ* to plant carbon balance and global carbon modeling, our understanding of and ability to measure these 2 parameters quickly and efficiently across a wide variety of environmental conditions remains a challenge.

*R*_L_ is a small but crucial flux of CO_2_ from the leaf (Tcherkez et al. 2017b). It forms an integral component of leaf carbon budgets, as the sum of *R*_L_, photosynthesis, photorespiration, and respiration in the dark (*R*_D_) ultimately determines a leaf's carbon balance (Heskel et al. 2013a). Thus, it is imperative that we accurately quantify *R*_L_ to gain robust carbon balance estimates. Evidence suggests that respiration differs between illuminated and nonilluminated leaves, with illumination resulting in the suppression of respiration, such that *R*_L_ is frequently lower than *R*_D_ (Tcherkez et al. 2017a). Nevertheless, *R*_L_ has been reported to be anywhere from 16 to over 100% of *R*_D_ (Hurry et al. 1996, 2005; Kroner and Way 2016; Gong et al. 2018; Way et al. 2019). The conflicting findings and paucity of data on *R*_L_ have led to *R*_L_ being represented as a fixed proportion of *R*_D_ in ecosystem models (Thornley and Cannell 2000; Malhi et al. 2015; Huntingford et al. 2017). Additional work is urgently needed to represent this flux with greater accuracy.

The large variability in estimates of *R*_L_ highlights one of the main challenges associated with understanding this flux—namely that *R*_L_ occurs concurrently with CO_2_ release via photorespiration and CO_2_ uptake via photosynthesis (Tcherkez et al. 2012a, 2017b). The interconnected nature of these pathways has made it challenging to identify the source of *R*_L_. Currently, *R*_L_ is thought to be the result of CO_2_ released by several processes. These include the decarboxylation reactions associated with the TCA cycle (Tcherkez et al. 2012b; Griffin and Heskel 2013; Tcherkez and Atkin 2021), the decarboxylation of pyruvate for fatty acid synthesis (Williams and Randall 1979), and the conversion of glucose 6-phosphate to ribose 5-phosphate through the oxidative pentose phosphate pathway or glucose 6-phosphate shunt (Sharkey and Weise 2016; Sharkey et al. 2020; Xu et al. 2021).

In addition to *R*_L_, accounting for the occurrence of photorespiration in global carbon models makes Γ* an important parameter in photosynthetic modeling (von Caemmerer 2000). Theoretically, Γ* describes the Rubisco CO_2_ compensation point and, thus, integrates the affinity of rubisco for CO_2_ or O_2_ with the stoichiometric release of CO_2_ per rubisco oxygenation. More specifically, it describes the CO_2_ concentration in the chloroplast when the oxygenation velocity of the enzyme is twice its carboxylation velocity. Despite its importance for modeling photosynthesis, we cannot measure Γ* directly. Instead, Γ* is usually estimated by measuring *C*_i_*, the CO_2_ concentration in the intercellular air space when Rubisco's oxygenation velocity is inferred to be twice its carboxylation velocity, and calculating the chloroplastic CO_2_ concentration according to


(1) $$\Gamma^{*}=C_{i}^{*}+\frac{R_{L}}{g_{m}}$$

where *g_m_* is mesophyll conductance (von Caemmerer 2000). From this equation, we can see how the estimation of Γ* depends on the estimation of *R*_L_.

Measurements of *R*_L_ and *C*_i_* can be made with gas exchange methods; however, these are time consuming, taking anywhere from 45 min to over an hour, limiting our ability to measure these parameters across a wide range of species and environmental conditions. Both *R*_L_ and *C*_i_* can be measured using the Laisk method (Laisk 1977). The Laisk method requires measuring the response of net CO_2_ assimilation (*A*) to changes in intercellular CO_2_ concentrations (*A/C*_i_ curves) at 3 or more different light levels (Laisk 1977; Atkin et al. 2000). This method assumes that the ratio of carboxylation (*v*_c_) and oxygenation (*v*_o_) and *R*_L_ are independent of light (Laisk 2022), leading to the intersection of the curves at a single point where *A* equals –*R*_L_ and *C*_i_ is *C*_i_*. By using Equation 1 and correcting for diffusion resistance through the mesophyll (i.e. *g*_m_), Γ* is estimated. While both these parameters can be measured using the Laisk technique, the time-consuming nature of the Laisk technique means that ecophysiological studies frequently measure only *R*_L_ using the Kok method (Kok 1948) or the Yin method (Yin et al. 2009, 2011). In both these methods, *R*_L_ is estimated from the response of *A* to irradiance. Around the light compensation point (approximately 10 to 40 *µ*mol photons m^−2^ s^−1^), a subtle shift in the slope of the light response of photosynthesis is apparent; the point where this shift occurs is called the breakpoint. Referred to as the “Kok effect,” this shift in slope has been interpreted to represent the suppression of leaf respiration by light (Kok 1948; Tcherkez et al. 2017a). *R*_L_ is estimated from the y-intercept of the linear fit of the points above the breakpoint, while *R*_D_ is estimated from the y-intercept of the linear fit of the points below the breakpoint (Heskel et al. 2013b; Tcherkez et al. 2017a; Yin et al. 2020). More recently, a modification to the Kok method has been recommended because the Kok method assumes a constant photosystem II electron transport efficiency (Φ_2_) across all light levels (Yin et al. 2009, 2011). The Yin method incorporates the decline in Φ_2_ with light and leads to slightly higher estimates of *R*_L_ than the Kok method (Yin et al. 2011). Estimates of *R*_L_ from the Yin method are comparable to those of the Laisk method (Yin et al. 2011).

All 3 of these methods are commonly employed under the steady-state conditions usually required by commercial gas exchange systems. Here, we refer to steady-state conditions within the sample and reference channel gas analyzer and not necessarily a metabolic steady-state. These 3 methods require tens of minutes just above or below the CO_2_ and/or light compensation points in each of the methods, potentially causing changes in photosynthetic and/or respiratory metabolism. Much more rapid nonsteady-state gas exchange approaches are becoming available, such as the rapid *A/C*_i_ response technique, although this approach requires frequent blank chamber corrections (Stinziano et al. 2017; Taylor and Long 2019). More recently, a nonsteady-state gas exchange measurement approach, called the dynamic assimilation technique (DAT), has been successfully employed to collect rapid *A/C*_i_ curves without the need for frequent empty chamber corrections (Saathoff and Welles 2021), substantially reducing the time spent under negative carbon balance conditions. This method is based on the same fundamental CO_2_ mass balance used to calculate steady-state gas exchange measurements for a leaf chamber with a well-mixed continuous airflow, except the equations are resolved according to nonsteady-state assumptions (Saathoff and Welles 2021). This allows accurate assimilatory and transpiratory flux measurements to be made during a change in CO_2_ concentration, greatly increasing the speed at which an individual CO_2_ response can be measured. If this method is reliable, it will decrease the time required to collect Laisk measurements, while substantially increasing the amount of data available for curve fitting, allowing the Laisk approach to be taken into the field. Ultimately, it is possible that this combination may decrease the uncertainty in *R*_L_ and *C*_i_*, providing a more effective Laisk measurement protocol.

In 2 studies, we compared Laisk measurements using DAT to conventional gas exchange estimates of *R*_L_ and *C*_i_*. In both studies, we chose to focus on testing the efficacy of the DAT Laisk method, specifically in species using the C_3_ photosynthetic pathway. In the first study, we estimated *R*_L_ and *C*_i_* in paper birch (*Betula papyrifera*), a common boreal tree species, grown under a range of elevated temperature and CO_2_ concentration conditions. Boreal forests are expected to experience warming of up to 9 °C by 2100 as a result of anthropogenic climate change (Collins et al. 2013). There are few datasets of *R*_L_ in boreal species (but see Kroner and Way 2016). Furthermore, the few studies examining temperature and/or CO_2_ effects on *R*_L_ show contrasting results. In some studies, long-term warming leads to thermal acclimation of *R*_L_ (Atkin et al. 2006; Heskel et al. 2013a; Kroner and Way 2016), while, in others, it has little effect on *R*_L_ (Way et al. 2019). Similarly, elevated growth CO_2_ concentrations can lead to an increase (Wang et al. 2001), no change (Ayub et al. 2011; Kroner and Way 2016), or a decrease in *R*_L_ (Ayub et al. 2014). Our measurements of *R*_L_ and *C*_i_* in paper birch allowed us to assess the acclimation potential of *R*_L_ in this species. At the same time, the diverse growth conditions provided a wide range of environmental conditions over which to compare DAT and steady-state Laisk estimates under field-like conditions. We expected to find acclimation of *R*_L_ in response to warming, but no impact of elevated CO_2_ concentration, consistent with observations from another boreal species (Kroner and Way 2016). We also hypothesized that DAT and steady-state estimates of *R*_L_ and *C*_i_* would be comparable.

In the second study, we tested the efficacy of DAT Laisk against the Yin method for estimating *R*_L_ in hybrid poplar (*Populus nigra* L. × *P. maximowiczii* A. Henry “NM6”) exposed to high or low CO_2_ concentration pre-treatments. This experiment was prompted by observations of subtle differences in *C*_i_* between DAT and steady-state methods in the first study with *B. papyrifera*. As described above, Γ* reflects intrinsic Rubisco kinetic parameters and thus should theoretically be fixed at any given O_2_ partial pressure. Given our findings in *B. papyrifera*, our goal was to explore whether differences in intercellular CO_2_ concentrations immediately prior to making measurements are responsible for differences in *C*_i_* between DAT and steady-state methods. We hypothesized that pre-treating with high and low CO_2_ concentrations before making the DAT Laisk measurements would further exaggerate the differences we found between *C*_i_* collected via the steady-state and DAT Laisk methods in paper birch. Ultimately, our goals across the 2 studies were 2-fold; first, to test the efficacy of the DAT Laisk method for measuring *R*_L_ and *C*_i_*, and second, to provide information on the impacts of future climate conditions on *R*_L_ for an important boreal species.

## Results

### Experiment 1

#### Experimental treatments

The 3 temperature treatments were successful in maintaining target temperature differences of +4 °C (T4) or +8 °C (T8) from the ambient temperature treatment (T0). During the summer season, maximum temperatures in each of the temperature treatments were 37 °C (T8), 33 °C (T4), and 29 °C (T0) (Supplemental Fig. S1A). Ambient and elevated CO_2_ concentration treatments were also successfully maintained with mean CO_2_ concentrations of 398 ± 54 ppm and 739 ± 48 ppm (means ± Sd) in the ambient concentration (AC) and elevated concentration (EC) glasshouses, respectively (Supplemental Fig. S1B).

#### Gas exchange

**Figure 1.**
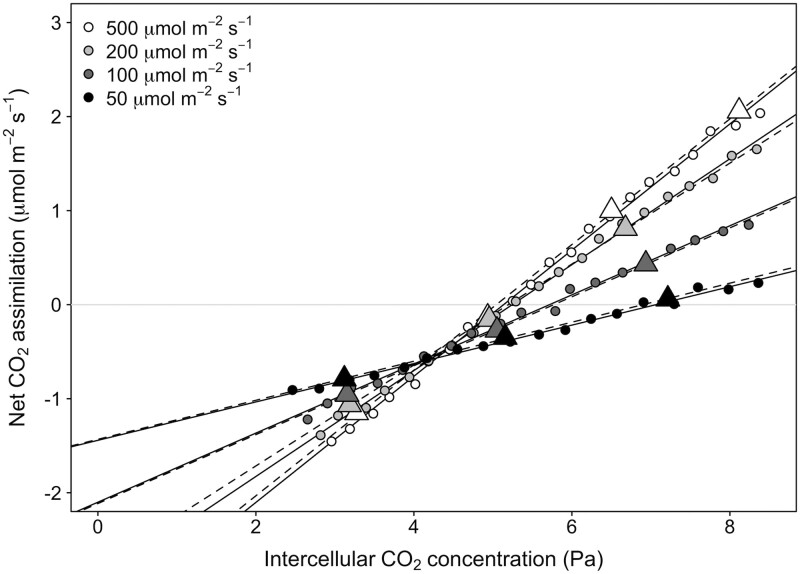
Representative data used to estimate *R*_L_ using the Laisk method. The response of net CO_2_ assimilation to intercellular CO_2_ was measured at 4 different irradiances taken both with the DAT Laisk method (Laisk_DAT_; circles) or the steady-state Laisk method (Laisk_SS_; triangles). Linear fits to the datapoints are shown, with Laisk_DAT_ as solid lines and Laisk_SS_ as dashed lines.

Example data used to calculate *R*_L_ and *C*_i_* using the steady-state and the DAT Laisk methods are shown in Fig. 1. The DAT Laisk method generated a much larger number of data points at each light level from which to identify the intersection point. Additionally, DAT Laisk curves were completed in approximately 25 min, while steady-state measurements took on average 100 min (Supplemental Fig. S2). Linear regressions of the initial slopes of the *A/C*_i_ curves for both showed excellent fits to the data across all irradiances under the different treatments (*r^2^* > 0.96 for both methods). DAT resulted in less uncertainty in the slope and intercepts of the linear regressions at each light intensity across the treatments (slope Ses ranged from 0.0027 to 0.0155 for Laisk_DAT_ and 0.0001 to 0.0506 for the Laisk_SS_; intercept Ses ranged from 0.0150 to 0.0950 for Laisk_DAT_ and 0.0060 to 0.2808 for Laisk_SS_). Even so, the uncertainty across both methods was very small, and there was little difference in the uncertainty in the intersection point and the parameters *R*_L_ and *C*_i_* derived from the slope-intercept regression method (*r^2^* > 0.99 for both methods; *R*_L_  Ses ranged from 0.006 to 0.148 for Laisk_DAT_ and 0.006 to 0.073 for Laisk_SS_, and *C*_i_* Ses ranged from 0.012 to 0.268 for Laisk_DAT_ and 0.013 to 0.153 for Laisk_SS_).

**Figure 2.**
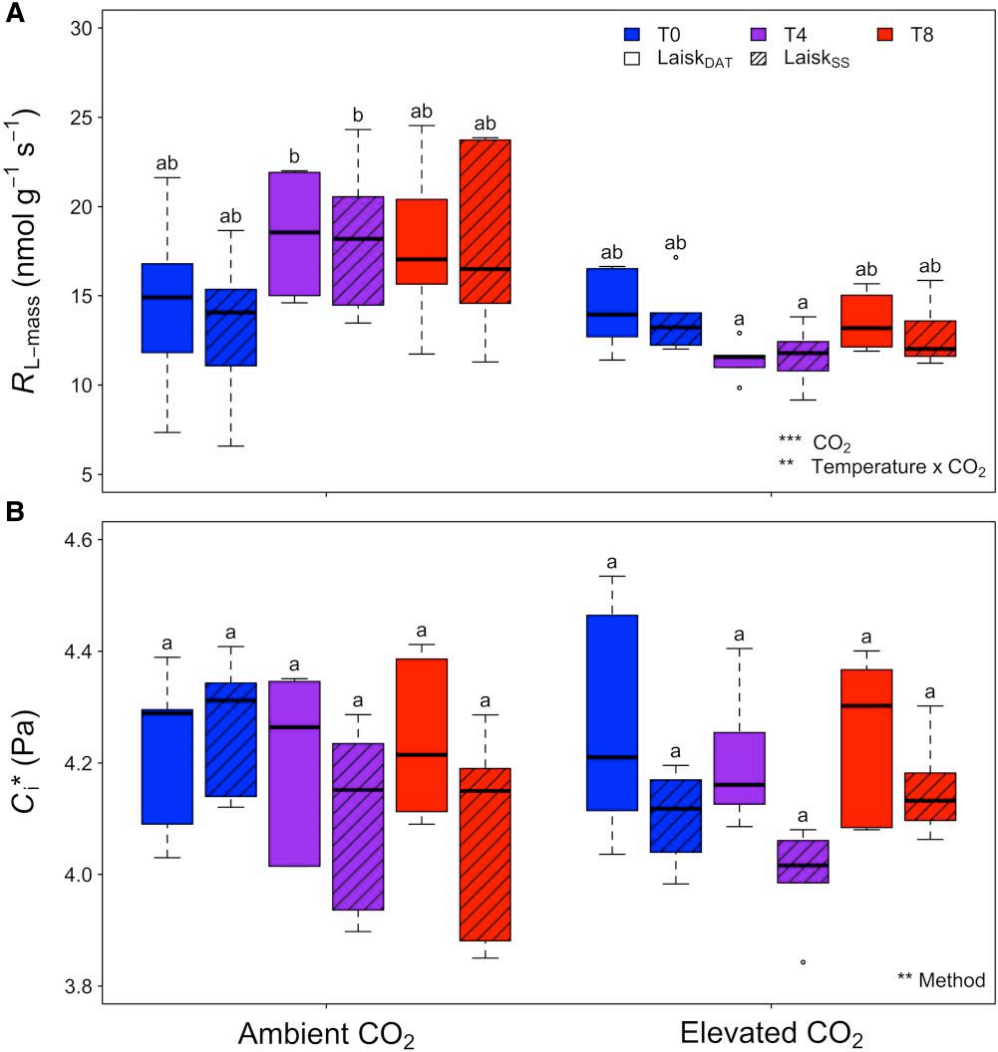
Traits estimated from the DAT and steady-state Laisk methods (Laisk_DAT_ or Laisk_SS_, respectively) on paper birch (*Betula papyrifera*) from each of the 6 environmental treatments. **A)** Mass-based respiration in the light (*R*_L-mass_) and **B)**  *C*_i_*. Colors represent the temperature treatments, with ambient temperature (T0) in blue (boxplots 1 & 2 in each CO_2_ treatment), ambient +4 °C (T4) in purple (boxplots 3 & 4 in each CO_2_ treatment), and ambient +8 °C (T8) in red (boxplots 5 & 6 in each CO_2_ treatment). Hatching denotes measurement method (either hatched to represent Laisk_DAT_, or not, representing Laisk_SS_). The boxplots represent the median as well as the first and third quartiles. Whiskers delimit the range for each group, with outliers falling outside 1.5 × the interquartile range marked by points. Significance of the main effects for each trait as determined by a 3-way ANOVA are noted (***P < 0.001; **P < 0.01). Full ANOVA results are found in Table 1. Different letters denote significant pairwise differences (*P* < 0.05) as determined via a Tukey post hoc comparison.

**Figure 3.**
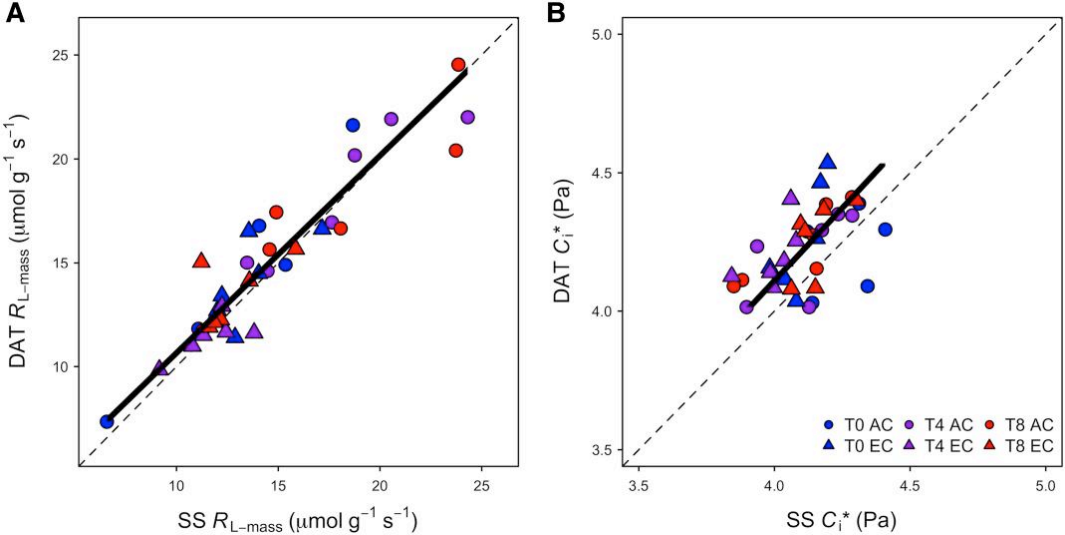
1:1 comparisons of traits estimated from DAT and steady-state (SS) Laisk methods on paper birch (*Betula papyrifera*). **A)**  *R*_L-mass_ and **B)**  *C*_i_*. Colors represent the temperature treatments, with ambient temperature (T0) in blue, ambient +4 °C (T4) in purple, and ambient +8 °C (T8) in red. Circles denote the ambient CO_2_ treatments (AC) and triangles the elevated CO_2_ treatments (EC). The dashed line shows the 1:1 line.

Across treatments, no significant differences in mass-, area-, or per N-based *R*_L_ (*R*_L-mass_, *R*_L-area_, *R*_L-N_, respectively) were apparent between the measurement methods (Fig. 2A, Table 1; Supplemental Fig. S3; Supplemental Table S1). There was a strong significant relationship between *R*_L-mass_ measured with the steady-state method and *R*_L-mass_ measured with the DAT method (Table 2), and the slope of this regression was not significantly different than the 1:1 line (Fig. 3A, Table 2). Consistency between the 2 Laisk methods was also apparent when *R*_L_ was examined on a leaf area basis (Supplemental Fig. S4, Supplemental Table S2). In contrast, the results for *C*_i_* were less consistent. The major axis regression comparing the DAT and steady-state estimates of *C*_i_* showed no significant difference from the 1:1 line (Fig. 3B, Table 2). However, the measurement method was the only significant predictor of differences in *C*_i_* in the ANOVA test, even though the Tukey post hoc test showed no significant pairwise differences. This is because there was large variability across the measurements; however, Laisk_DAT_  *C*_i_* estimates were consistently larger than those of the steady-state method (Fig. 2B, Table 1).

**Table 1.** Summary of the 3-way ANOVAs in paper birch (*Betula papyrifera*)

Effects	*R*_L-mass_		*C*_i_*	
	*F*-value	*P*-value	*F*-value	*P*-value
Temperature	1.277	0.287	1.973	0.148
CO_2_	23.679	**<0**.**001**	0.536	0.467
Method	0.286	0.595	12.018	**0**.**001**
Temperature: CO_2_	6.288	**0**.**003**	1.042	0.359
Temperature: method	0.102	0.903	0.559	0.575
CO_2_: method	0.007	0.935	1.463	0.231
Temperature: CO_2_: method	0.092	0.912	1.186	0.313

*F*-values and *P-*values with temperature, CO_2_, and measurement method as the main effects are shown. Traits analyzed were mass-based respiration in the light (*R*_L-mass_) and *C*_i_*. Bold numbers represent *P* < 0.05.

**Table 2.** Linear regressions of the 2 Laisk methods, DAT and steady-state, used to estimate mass-based respiration in the light (*R*_L-mass_) and *C*_i_* in paper birch (*Betula papyrifera*)

Parameter	*n*	*P-*value	*R* ^2^	Slope	95% CI	H0 #1 slope = 1	Intercept	95% CI	H0 #2 intercept = 0
*R*_L-mass_	35	<0.001	0.86	0.951	(0.824, 1.096)	0.472	1.147	(−0.880 to 3.175)	0.258
*C*_i_*	35	0.001	0.28	1.052	(0.556, 2.038)	0.857	−0.099	(−2.574 to 2.376)	0.936

A statistically similar slope (*P* > 0.05) indicates no significant differences in the 2 methods. Number of samples (*n*), significance values (*P-*values), coefficients of determination (*R*^2^), slope parameter estimates (slope), 95% confidence intervals (95% CI), test of whether the slope equals 1 (H0 #1 slope = 1), intercept parameter estimates (intercept), and test of whether the intercept equals 0 (H0 #2 intercept = 0).

Measurements of *R*_L_ across the treatments not only provided a strong test of the DAT Laisk method they also furnished a unique opportunity to assess acclimation of *R*_L_ to elevated temperature and CO_2_ in *B. papyrifera.* This boreal species showed only small evidence of thermal acclimation in *R*_L-mass_. Moreover, while CO_2_ treatment and the interaction between CO_2_ and temperature were found to be significant predictors, the only notable difference according to the Tukey post hoc analysis was a decrease in *R*_L-mass_ between ambient and elevated CO_2_ in the +4 temperature treatment (Fig. 2A, Table 1). Both temperature and CO_2_ were significant predictors of R_L-area_, likely reflecting a trend toward higher *R*_L_ in T4 compared to T0 or T8 seedlings and a trend toward higher *R*_L_ with elevated CO_2_ (Supplemental Fig. S3A, Supplemental Table S1). Nevertheless, few statistical differences were found in the post hoc analysis. Similarly, temperature, CO_2_, and their interaction were significant predictors for *R*_L-N_, but again, pairwise comparisons showed few differences between treatments (Supplemental Fig. S3B, Supplemental Table S1).

#### Leaf traits

**Figure 4.**
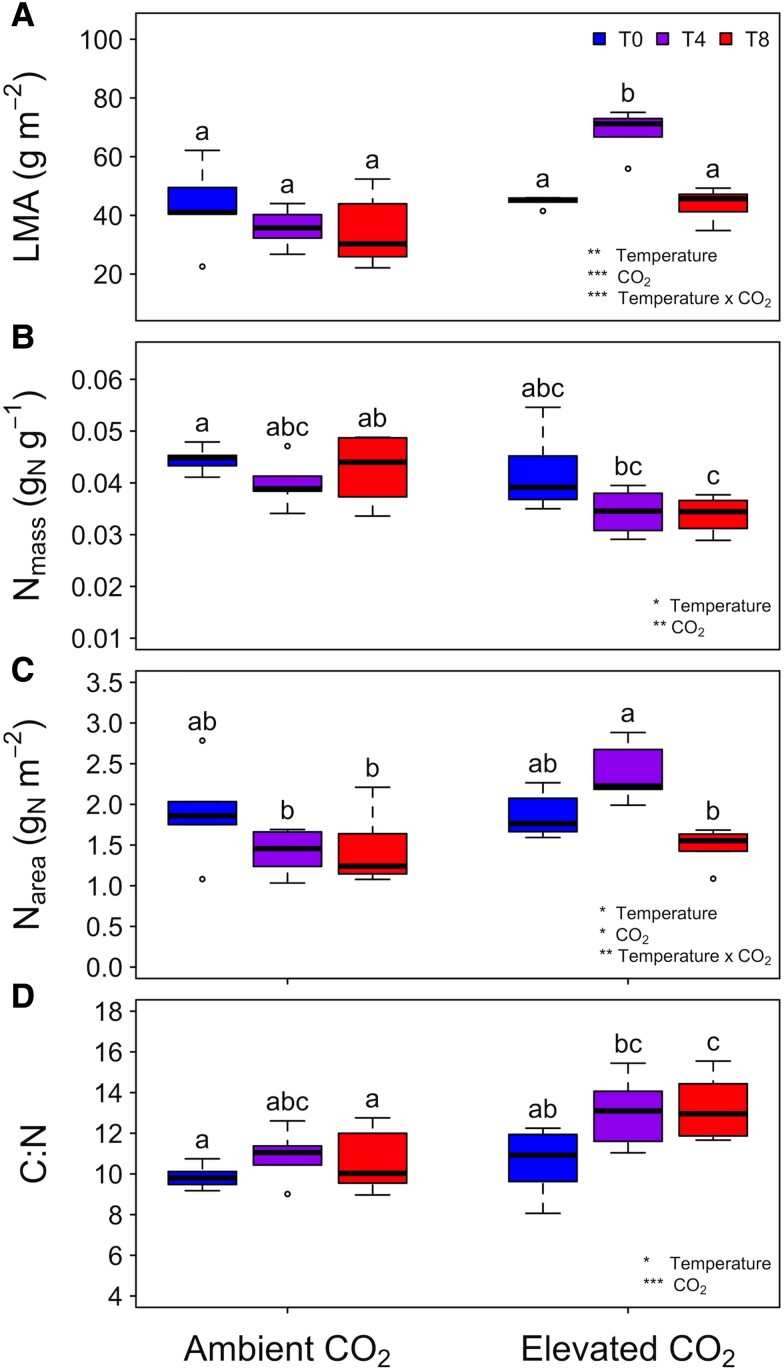
Leaf traits, including LMA, N_mass_, N_area_, and C:N of paper birch (*Betula papyrifera*), grown under the 6 environmental treatments. Colors represent the temperature treatments, with ambient temperature (T0) in blue (boxplot 1 in each CO_2_ treatment), ambient +4 °C (T4) in purple (boxplot 2 in each CO_2_ treatment), and ambient +8 °C (T8) in red (boxplot 3 in each CO_2_ treatment). The boxplots represent the median as well as the first and third quartiles. Whiskers delimit the range for each group, with outliers falling outside 1.5 × the interquartile range marked by points. Significance of the main effects for each trait as determined by a 2-way ANOVA are noted (***P < 0.001; **P < 0.01; *P < 0.05). Full ANOVA results are found in Table 3. Different letters denote significant pairwise differences (*P* < 0.05) as determined via a Tukey post hoc comparison.

Across treatments, there were some important differences in leaf traits (Fig. 4, Table 3). Leaf mass per area (LMA) was significantly higher in the ECT4 treatment compared to all other treatments (Fig. 4A, Table 3). Leaf mass-based nitrogen (N_mass_) tended to decrease with increasing temperature and at elevated CO_2_, although the only significant pairwise difference was between ACT8 and ECT8 trees (Fig. 4B, Table 3). Area-based nitrogen followed patterns found in LMA, with higher area-based nitrogen (N_area_) in the ECT4 seedlings (Fig. 4C, Table 3). Finally, the carbon to nitrogen ratio (C:N) tended to increase from ambient to elevated CO_2_, and with warming under elevated CO_2_ conditions (Fig. 4D, Table 3).

**Table 3.** Summary of the 2-way ANOVAs in *B*. *papyrifera* showing *F*-values and *P-*values with temperature and CO_2_ as the main effects

Leaf trait	*F*-value	*P*-value
**LMA**		
Temperature	7.396	**0**.**003**
CO_2_	27.808	**<0**.**001**
Temperature: CO_2_	10.629	**<0**.**001**
**N_mass_**		
Temperature	4.342	**0**.**022**
CO_2_	11.728	**0**.**002**
Temperature: CO_2_	1.059	0.360
**N_area_**		
Temperature	5.326	**0**.**011**
CO_2_	7.049	**0**.**013**
Temperature: CO_2_	6.359	**0**.**005**
**C:N**		
Temperature	5.355	**0**.**010**
CO_2_	15.953	**<0**.**001**
Temperature: CO_2_	1.424	0.257

Traits analyzed were LMA, N_mass_, N_area_, and C:N. Bold numbers represent *P* < 0.05.

### Experiment 2

**Figure 5.**
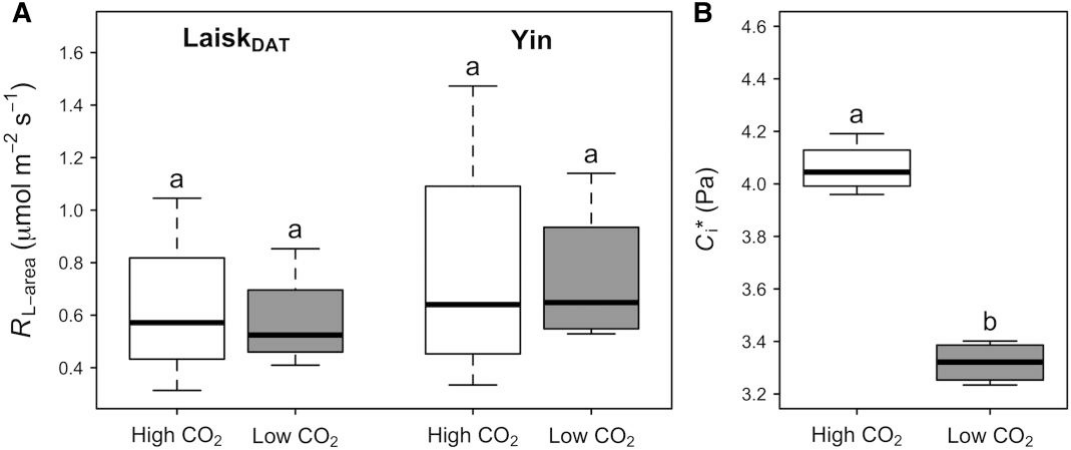
Traits measured on leaves of hybrid poplar (*Populus nigra × maximowiczii*) exposed to either high CO_2_ (1000 ppm; white) or low CO_2_ (25 ppm; gray) pre-treatments. **A)** Area-based respiration in the light (*R*_L-area_) estimated according to the DAT Laisk or Yin methods, and **B)**  *C*_i_* estimated according to the DAT Laisk method. The boxplots represent the median as well as the first and third quartiles. Whiskers delimit the range for each group, with outliers falling outside 1.5 × the interquartile range marked by points. Different letters denote significant differences (*P* < 0.05) between treatments as determined by a 2-way ANOVA and Tukey post hoc comparison for *R*_L-area_ and a t-test for *C*_i_*. *n* = 4.

Values for *C*_i_* differed more between Laisk_DAT_ and Laisk_SS_ than expected based on the results from Experiment 1. To determine whether these results could be replicated and exaggerated, we performed a second experiment in which leaves of *P. nigra × maximowiczii* were exposed to high or low CO_2_ concentrations before taking measurements of *R*_L_ and *C*_i_* using Laisk_DAT_ and the Yin methods. No significant differences in *R*_L_ were observed between the 2 methods (Laisk_DAT_ and Yin; Fig. 5A; *P* > 0.05). Furthermore, the 2 pre-treatments resulted in similar patterns to those found in the methods comparison in *B. papyrifera*. Specifically, no significant differences were found in *R*_L-area_ between the high and low CO_2_ pre-treatments (Fig. 5A; *P >* 0.05). However, significantly higher *C*_i_* was observed in the high CO_2_ compared to the low CO_2_ pre-treatment (Fig. 5B; *P =* 0.00002), following the same directional shift in *C*_i_* observed when comparing Laisk_DAT_ to Laisk_SS_ in *B. papyrifera* (higher *C*_i_* in Laisk_DAT_ compared to Laisk_SS_).

*A/C*_i_ curves were analyzed to obtain estimates of *g*_m_ under the high and low CO_2_ pre-treatments. In the high CO_2_ pre-treatment, *g*_m_ was 0.95 ± 0.15 *µ*mol m^−2^ s^−1^ Pa^−1^ and in the low CO_2_ pre-treatment, *g*_m_ was 0.80 ± 0.26 *µ*mol m^−2^ s^−1^ Pa^−1^. For both treatments, estimated *g*_m_ values represent means ± Se, *n* = 4.

## Discussion

We examined *R*_L_ in 2 species under different experimental conditions using both traditional steady-state measurement techniques and a DAT method made possible by improved gas exchange equations and stability of modern gas exchange systems such as the LI-COR 6800 (Saathoff and Welles 2021). Our findings demonstrate the reliability of DAT for measuring *R*_L_ using the Laisk method. Furthermore, comparing the faster DAT method to the steady state method and across CO_2_ pre-treatments exposed shifts in *C*_i_*. Finally, the lack of acclimation of *R*_L_ in paper birch has ramifications for the future ecology of this important boreal species. We discuss the implications of each of these findings in greater detail below.

### Insights from comparing DAT and steady-state Laisk methods

Comparisons of *R*_L_ values between Laisk_DAT_ and Laisk_SS_ in paper birch show equivalent parameter estimates in plants exposed to a variety of different temperature and CO_2_ growth conditions. Additionally, estimates of *R*_L_ in hybrid poplar were found to be similar when estimated via Laisk_DAT_ and the Yin method. Together, these findings from different species across varying growth conditions, and employing several methods for estimating *R*_L_, suggest that Laisk_DAT_ is a robust method for estimating *R*_L._ In contrast, *C*_i_* values were not consistently comparable between DAT and steady-state Laisk methods. The contrasting findings for *R*_L_ and *C*_i_* point to several advantages of using DAT and highlight areas that may require additional research.

One clear advantage to using DAT over steady-state methods for Laisk measurements is that Laisk_DAT_ can be completed 4 times faster than the steady-state method, reducing the time from 100 to 25 min (Supplemental Fig. S2). Thus, DAT provides an alternate method that gives comparable estimates of R_L_ while greatly decreasing the time needed to complete the measurements. DAT not only improves measurement efficiency but also shortens the time spent at low CO_2_ partial pressures compared to steady-state Laisk measurements. Remaining at very low CO_2_ partial pressures around the CO_2_ compensation point can cause the deactivation of Rubisco (von Caemmerer and Edmondson 1986; Walker and Ort 2015), which may in turn lead to a shift in the intersection point and thus parameter estimates. Since the use of DAT and CO_2_ ramps only requires 3 min for each CO_2_ response curve, the deactivation of Rubisco is less likely.

An additional potential advantage of Laisk_DAT_ is that the number of datapoints collected is much greater than the 4 to 6 datapoints typically collected from Laisk_SS_. Saathoff and Welles (2021) suggested that this may reduce uncertainty in identifying the crossover point and estimating *C*_i_*. While the greater density of points from the DAT method leads to more robust fits of the linear regressions at each light intensity, there is little difference in the uncertainty around the intersection point and *R*_L_ and *C*_i_* values derived from the slope-intercept regression analysis method. Thus, the main advantages of DAT over steady-state come from the increased speed of measurements, the removal of potential measurement artifacts related to Rubisco deactivation at low CO_2_ partial pressures, and other changes in physiology such as post-translational regulation of enzyme activity and changes in pH and ion concentrations as a result of ion movements across membranes (Li et al. 2021).

While there are many advantages to using DAT with Laisk measurements, several findings should be further explored. The most puzzling of these is the subtle but consistent increase in *C*_i_* from the steady-state to the DAT Laisk method in paper birch, a pattern that is also apparent when leaves of a different species, hybrid poplar, are exposed to high vs. low CO_2_ pre-treatments. The *C*_i_* is closely related to Γ* (the Rubisco compensation point) or the CO_2_ concentration at which the carboxylation velocity is half the oxygenation velocity (Brooks and Farquhar 1985; Laisk 2022). Since Γ* is partially a function of Rubisco kinetics, theoretically, it should be invariant within a species or growth treatment, assuming a constant stoichiometric release of CO_2_ per Rubisco oxygenation. It is unclear why we find consistent shifts in *C*_i_* between measurement methods and CO_2_ pre-treatments. However, we propose several possible explanations.

One possibility is that shifts in *C*_i_* result because of changes in mesophyll conductance (*g*_m_). *C*_i_* is defined as Γ* minus the CO_2_ from *R*_L_ diffusing from the intercellular airspace into the mesophyll (*g*_m_) (see Equation 1). If Γ* is fixed as expected from Rubisco kinetics and *R*_L_ is stable as seen in both of our experiments, then it follows that any changes in *C*_i_* could be the result of changes in *g*_m_. In our second experiment exploring the effect of CO_2_ pre-treatment on *C*_i_*, we also collected *A/C*_i_ curves to estimate *g*_m_. We found that *g*_m_ only varied by 0.15 *µ*mol m^−2^ s^−1^ Pa^−1^ between the high and low CO_2_ pre-treatments. If Γ* is fixed at 4.5 Pa and *R*_L_ is assumed to be the average found from the Laisk and Yin methods (∼0.67 *µ*mol m^−2^ s^−1^), then a 1.06 *µ*mol m^−2^ s^−1^ Pa^−1^ difference in *g*_m_ would be required to explain the observed differences in *C*_i_* with higher *g*_m_ in the high CO_2_ pre-treatment. Existing literature showing variation in *g*_m_ reports that low CO_2_ concentrations result in higher *g*_m_ (e.g. Flexas et al. 2007), which is the opposite direction needed to explain our *C*_i_* results. Since neither the magnitude nor the direction of the differences in *g*_m_ can explain our results, we conclude that *g*_m_ is unlikely to explain the differences found in *C*_i_* between pre-treatments.

An alternate possibility is that the variability in *C*_i_* may depend on how much CO_2_ is stoichiometrically released during photorespiration following Rubisco oxygenation. Commonly, it is assumed that 1 molecule of CO_2_ is released for every 2 oxygenation events; however, there are conditions where this ratio may vary. The best known of these is the dependence of photorespiratory CO_2_ release on amino acid export from the photorespiratory pathway. Busch et al. (2018) explore the consequences if either glycine or serine is removed from photorespiration. While there is growing consensus that serine export removes 30% to 40% of carbon from photorespiration (Busch et al. 2018; Fu et al. 2023), serine export does not alter the ratio of CO_2_ release per oxygenation (Supplemental Text S1, section 2). However, export of 10% of carbon via glycine from photorespiration can alter this ratio, leading to differences in *C*_i_* that are consistent with the 0.7 Pa difference found between high and low CO_2_ pre-treatments in hybrid poplar (Supplemental Text S1, section 2). These changes do not affect estimates of *R*_L_, which is also consistent with our experimental findings. Employing this dynamic technique to collect Laisk curves may have uncovered dynamic shifts in glycine export from photorespiration that could explain differences in *C*_i_* between pre-treatments in hybrid poplar and differences between DAT and steady-state measurements of *C*_i_* in paper birch. Additional work is needed to fully understand the mechanism leading to these consistent shifts in *C*_i_*. Nevertheless, it is clear from our experiments that *R*_L_ measurements taken with DAT are consistent with traditional steady-state methods, while reducing measurement artifacts.

### Ecological implications of *R*  _L_ estimates for paper birch

The first experiment on paper birch served not only to test the efficacy of DAT for estimating *R*_L_ but also to understand the impacts of climate change on a widespread boreal species. Overall, we demonstrate that there is little acclimation of *R*_L_ in this species when exposed to combinations of elevated temperatures and CO_2_. In this regard, our findings are most similar to 2 studies in *Eucalyptus* that found no significant effects of temperature or CO_2_ on *R*_L_ (Ayub et al. 2011; Crous et al. 2012, 2017). In contrast, a study on Norway spruce (*Picea abies*), a common Eurasian boreal tree, did show acclimation to temperature in *R*_L_ but no response to changes in CO_2_ concentration (Kroner and Way 2016). Together, these studies highlight that the response of *R*_L_ to changes in climate may be species specific, even if species hail from similar biomes (as is the case with paper birch and Norway spruce). Continued collection of the *R*_L_ response to elevated temperature and CO_2_ (in addition to photosynthesis and respiration) across species will therefore be important for a clear understanding of the climatic effects on this small but important plant carbon flux.

A closer comparison of our results on an area and a mass basis provides additional insight into mechanistic changes in paper birch in response to temperature in CO_2_ treatments. Most obvious in this comparison is the subtle decrease in *R*_L-mass_ and *R*_L-N_ in response to elevated CO_2_, but a contrasting increase in *R*_L-area_ when exposed to the +4 temperature and elevated CO_2_ treatment. These unit-dependent differences in *R*_L_ are a direct result of the higher LMA found in the ECT4 trees. Overall, the mass- and nitrogen-based findings imply a reduction in investment in *R*_L_ per unit of tissue or protein constructed in response to elevated CO_2_ and warming, a finding not seen on an area-basis. This emphasizes the importance of considering the units used to assess gas exchange when interpreting results from climate change studies as these unit-based differences could underlie some of the variation we see among studies in the literature (see references in Tcherkez et al. 2017b). Indeed, past work has also found contrasting area- and mass-based results in *R*_L_ (e.g. Ayub et al. 2014) leading to the conclusion that the interaction of environmental factors such as nitrogen availability and soil quality, in addition to temperature and CO_2_ concentrations, may ultimately drive CO_2_ efflux via *R*_L_ (Tcherkez et al. 2017b).

It is worth mentioning here that perhaps much of the confusion in understanding these unit-based differences and the effects of environmental interactions on *R*_L_ is because the source of *R*_L_ remains unclear. Thus, while studies have correlated *R*_L_ to *R*_D_, photorespiration, Rubisco carboxylation, and even nitrogen content (e.g. Ayub et al. 2014), we still do not know the ultimate source, or the role, of this small but important flux in the grand picture of a plant's carbon balance (Tcherkez et al. 2017b; Gauthier et al. 2020; Xu et al. 2021, 2022). The advent of this rapid DAT method provides an exciting opportunity to expand our understanding of *R*_L_ across a larger number of species and variable climate conditions.

## Materials and methods

### Experiment 1

#### Study design

Seeds of paper birch (*B. papyrifera* [Marshall]), a deciduous broad-leaved species, were sourced from Ontario between 45 and 46°N latitude. All seeds were sown in Pro-Mix BX Mycorrhizal growth medium (Premier Tech Home and Garden, QC, Canada) and slow-release fertilizer (Slow Release Plant Food, 12-4-8, Miracle Gro, The Scotts Company) in 11.6-L pots in early May 2021. Twenty replicate pots were placed into 6 glasshouses in the Biotron Experimental Climate Change Research Centre of Western University. Glasshouses were set to the following 6 factorial climate treatments: AC or EC CO_2_ (AC, ∼410 ppm; EC, ∼750 ppm, respectively, where ppm is defined as mole fraction) with 1 of 3 temperature treatments: ambient temperature (T0), ambient +4 °C (T4), or ambient +8 °C (T8). Ambient temperature conditions were set to a 5-year day/night average for Algonquin Park, ON (45°58′N, 78°48′W). Warming treatments were chosen to represent moderate and extreme climate scenarios for the same location (Collins *et al.* 2013). In all glasshouses, relative humidity was maintained above 60%, and irradiance tracked local ambient conditions with average photoperiod ranging from ∼15 h during the summer season to ∼12 h in October, and photosynthetic photon flux density (PPFD) ranging from 50 to 1300 *µ*mol m^−2^ s^−1^ (PPFD was calculated from solar radiation in W m^−2^ by multiplying by 2.02 [Mavi and Tupper 2004]). Elevated CO_2_ treatments were maintained by adding pure CO_2_ to the chambers until the elevated setpoint was reached. CO_2_, temperature, and relative humidity were controlled by Argus Control Software TITAN version 900 (Surrey, BC, Canada). All seedlings were watered as needed to prevent water stress, and soil moisture was periodically checked using a soil moisture probe (HH2 Moisture Meter, Delta-T Devices, Cambridge, UK) to ensure consistent soil moisture among treatments. Measurements of *R*_L_ and *C*_i_* were taken from mid-September to early October 2021.

#### Gas exchange measurements

Gas exchange measurements were taken on a single fully expanded sun leaf from 5 to 6 individuals of paper birch from each treatment using an LI-6800 with the 6 cm^2^ multiphase flash fluorometer chamber (LI-COR Biosciences, Lincoln, NE, USA). Dynamic assimilation equations were enabled on the LI-6800. Before taking measurements, the chamber and flow-rate specific tuning parameters for the dynamic assimilation calculations were measured using an empty chamber and a flow rate of 500 *µ*mol s^−1^, and a range match was determined across all experimental CO_2_ concentrations. Leaf temperature was set to 25 °C, and the relative humidity of the chamber air was controlled at 60%. Vapor pressure deficit (VPD) under these conditions was on average 1.2 kPa and ranged from 1.0 to 1.3 kPa. *R*_L_ and *C*_i_* were assessed by the Laisk method as described by Laisk (1977), in which curves of net photosynthesis vs. intercellular CO_2_ concentration (*A*/*C*_i_) are collected at 3 or more irradiances. Measurement irradiances for paper birch were 500, 200, 100, and 50 *µ*mol photons m^−2^ s^−1^. These light intensities were chosen based on preliminary measurements, which confirmed adequate differences in the slopes of the 4 *A/C*_i_ curves to result in a clear common intersection.

Two methods were employed to collect the *A/C*_i_ curves used to calculate *R*_L_ and *C*_i_*. In both methods, the initial cuvette CO_2_ concentration was set to 400 ppm until CO_2_ fluxes were stable. Then, in the first method (Laisk_SS_), traditional steady-state *A/C*_i_ curves were taken at each of the above light intensities at the following CO_2_ concentrations: 150, 100, 75, 50, 25, and then back to 400 ppm before starting the next curve. Leaves were left at each CO_2_ concentration for at least 30 s but no more than 120 s, for a total time at low CO_2_ (150 ppm and below) of 8 min per curve. In the second method (Laisk_DAT_), the DAT was employed. At each light intensity, the reference CO_2_ concentration was ramped from 150 to 0 ppm at a rate of 50 ppm min^−1^. During the ramp, measurements were logged every 5 s to provide high resolution response curves taking just 3 minutes for each light intensity. Some lag is inevitable between reference and sample CO_2_ concentrations when ramping the reference CO_2_. As a result, the lowest logged sample CO_2_ concentration reached across the DAT curves was 18.9 ± 0.0026 ppm (mean ± Se), just 6 ppm below the lowest CO_2_ concentration in Laisk_SS_. In *B. papyrifera*, both Laisk_SS_ and Laisk_DAT_ methods were compared and data from both methods were analyzed using points below a *C*_i_ of 8.45 Pa. *R*_L_ and *C*_i_* were estimated from both the Laisk_SS_ and Laisk_DAT_ curves according to the slope-intercept regression method (Walker and Ort 2015; Walker et al. 2016).

#### Leaf traits

After completing gas exchange measurements on *B. papyrifera*, leaves were dried at 60 °C to a constant mass. Leaf dry mass was measured, and LMA was calculated by dividing the leaf mass by the leaf area. LMA was used to convert area-based (*R*_L-area_) to mass-based (*R*_L-mass_) respiratory fluxes. Samples were then ground (Wiley Mill, Thomas Scientific, NJ, USA), and leaf carbon and nitrogen concentrations were measured using an elemental analyzer (vario ISOTOPE cube, Elementar, Germany). From the resulting percentages, we calculated the C:N, N_area_ and N_mass_, and respiration per unit of nitrogen (*R*_L-N_).

#### Statistical analyses

In Experiment 1, differences in *R*_L_ and *C*_i_* in paper birch between CO_2_ and temperature treatments and between Laisk measurement methods were assessed using a 3-way ANOVA with a Tukey post hoc test with a significance value of 0.05. All data were assessed to ensure they fulfilled assumptions of normality. Additionally, whether estimates of *R*_L_ and *C*_i_* were consistent between the DAT and steady-state measurement techniques was assessed by using major axis regression in the smatr package in R (Warton et al. 2012) and comparing the slopes of the regression lines between methods to a 1:1 line. All data analysis took place in R v. 4.1.3 (R Core Team 2022).

### Experiment 2

#### Study design

To explore possible causes of the subtle differences in *C*_i_* found between the DAT and steady-state Laisk methods in paper birch, an additional experiment was undertaken on saplings of hybrid poplar (*P. nigra* L. × *P. maximowiczii* A. Henry “NM6”). Continuously flushing plants were grown in the glasshouses of Michigan State University in 10-L pots of potting media consisting of 70% peat moss, 21% perlite, and 9% vermiculite (Suremix; Michigan Grower Products Inc., Galesburg, MI, USA). Typical daylight light levels were between 300 and 700 *µ*mol photons m^−2^ s^−1^ and daylength was extended to 16 h with high pressure sodium lamps. The daytime temperature was controlled to 27 °C during the day. Plants were watered with half-strength Hoagland's solution 3 times per week and reverse osmosis water on the other days. Before gas exchange measurements (described in the next section), a recently fully-expanded leaf near the top of the canopy was clamped in the fluorescence chamber of the LI-6800 (LI-COR Biosciences, Lincoln, NE, USA) and exposed to 1 of 2 pre-treatments for 45 min: high CO_2_ of 1000 ppm and high photosynthetically active radiation (PAR) of 1000 *µ*mol photons m^−2^ s^−1^, or low CO_2_ of 25 ppm and high PAR of 1000 *µ*mol photons m^−2^ s^−1^.

#### Gas exchange measurements

Gas exchange measurements were taken on a single fully-expanded sun leaf from 4 individuals of hybrid poplar from each treatment using an LI-6800 with the multiphase flash fluorometer chamber (LI-COR Biosciences, Lincoln, NE, USA). In this experiment, the DAT Laisk method was used (as in Experiment 1) to measure *R*_L_ and *C*_i_*. Briefly, dynamic equations were enabled on the LI-6800, dynamic tuning was tested using an empty chamber and a flow rate of 500 *µ*mol s^−1^ and range matching was employed. Leaf temperature was set to 25 °C. The reference H_2_O was set to 18 mmol mol^−1^. Laisk *A*/*C*_i_ curves were collected at 4 irradiances: 300, 125, 75, and 50 *µ*mol photons m^−2^ s^−1^. Once again, light intensities were chosen based on preliminary measurements, which confirmed adequate differences in the slopes of the 4 *A/C*_i_ curves. At each light intensity, reference CO_2_ concentrations were ramped from 150 to 0 ppm at a rate of 50 ppm min^−1^ with data logged every 5 s. Data were subset to the points below a *C*_i_ of 8.45 Pa, and *R*_L_ and *C*_i_* were estimated according to the slope-intercept regression method (Walker and Ort 2015; Walker et al. 2016).

These measurements of *R*_L_ via Laisk_DAT_ were compared to *R*_L_ estimated via another common measurement technique, the Yin method (Yin et al. 2009, 2011). For these measurements, simultaneous steady-state gas exchange and chlorophyll fluorescence measurements were taken with the LI-6800 at the following light intensities: 100, 75, 50, 45, 40, 35, 30, 25, 20, 15, 10, and 5 *µ*mol photons m^−2^ s^−1^. Fluorescence data were used to calculate photosystem II electron transport efficiency ($\Phi_{2}$) at each light intensity as $1-F_{s}/F_{m}^{\prime }$. The increase in $\Phi_{2}$ at low light levels was then incorporated into the light response curve by plotting *A* as a function of *I*_inc_  $\Phi_{2}/4$ , where *I*_inc_ is the incident irradiance. Finally, linear regression of *A* vs. *I*_inc_  $\Phi_{2}/4$ was used to determine the intercept or *R*_L_. A full theoretical derivation of the technique is available in Yin et al. (2009) and Yin et al. (2011).

After collecting the Laisk and Yin curves, a full *A/C*_i_ curve was collected using DAT. CO_2_ was ramped from 1,000 to 0 ppm at an irradiance of 200 *µ*mol photons m^−2^ s^−1^ and a rate of 300 ppm min^−1^ with data logged every 5 s. Flow rate was set to 500 *µ*mol s^−1^, leaf temperature to 25 °C, and reference H_2_O to 18 mmol mol^−1^. A low irradiance was used to improve fitting capabilities for mesophyll conductance (*g*_m_). *A/C*_i_ curves were analyzed with an updated version of the spreadsheet from Sharkey (2016), which incorporates the equations from Busch et al. (2018) using a fixed value of *R*_L_ of 0.67 *µ*mol m^−2^ s^−1^ (the average of *R*_L_ estimates acquired from DAT Laisk and Yin methods).

### Statistical analyses

In Experiment 2, differences in *R*_L_ among pre-treatments (high or low CO_2_) and method (Laisk_DAT_ or Yin) were assessed using a 2-way ANOVA and Tukey post hoc test. Differences in *C*_i_* between the 2 CO_2_ pre-treatments were compared using a *t*-test. All data analysis took place in R v. 4.1.3 (R Core Team 2022).

## Supplementary Material

kiad305_Supplementary_Data

## Data Availability

Data are archived with Dryad digital repository: https://doi.org/10.5061/dryad.2v6wwpztk (Schmiege et al. 2023).

## Supplemental data

The following materials are available in the online version of this article.

**Supplemental Figure S1.** Environmental treatments in each of the six glasshouses for the duration of experiment 1.

**Supplemental Figure S2.** Total time taken to perform the DAT compared to the steady-state Laisk method in paper birch (*Betula papyrifera*).

**Supplemental Figure S3.** Respiration in the light estimated according to the DAT and the steady-state Laisk methods (Laisk_DAT_ or Laisk_SS_; respectively) on paper birch (*Betula papyrifera*) from each of the six environmental treatments.

**Supplemental Figure S4.** 1:1 comparison of *R*_L-area_ calculated according to the DAT and the steady-state (SS) Laisk methods on paper birch (*Betula papyrifera*).

**Supplemental Table S1.** Summary of the three-way ANOVA in paper birch (*B*. *papyrifera*).

**Supplemental Table S2.** Linear regression of the two methods, DAT and steady-state, used to estimate area-based respiration in the light (*R*_L-area_) in paper birch (*Betula papyrifera*).

**Supplemental Text S1.** Graphical methods for determining respiration in the light and the Rubisco compensation point.
