# Supplementary material for: Laisk measurements in the nonsteady state: Tests in plants exposed to warming and variable CO2 concentrations
Source: Plant Physiol. 2023 May 26;193(2):1045–57. doi: 10.1093/plphys/kiad305 (PMC10517191; doi:10.1093/plphys/kiad305)
Supplement: kiad305_Supplementary_Data [file kiad305_supplementary_data.pdf]

SUPPLEMENTAL INFORMATION

Laisk measurements in the non-steady-state: tests in plants exposed to warming and variable CO_2 concentrations

Stephanie C. Schmiege, Thomas D. Sharkey, Berkley Walker, Julia Hammer, Danielle A. Way

---

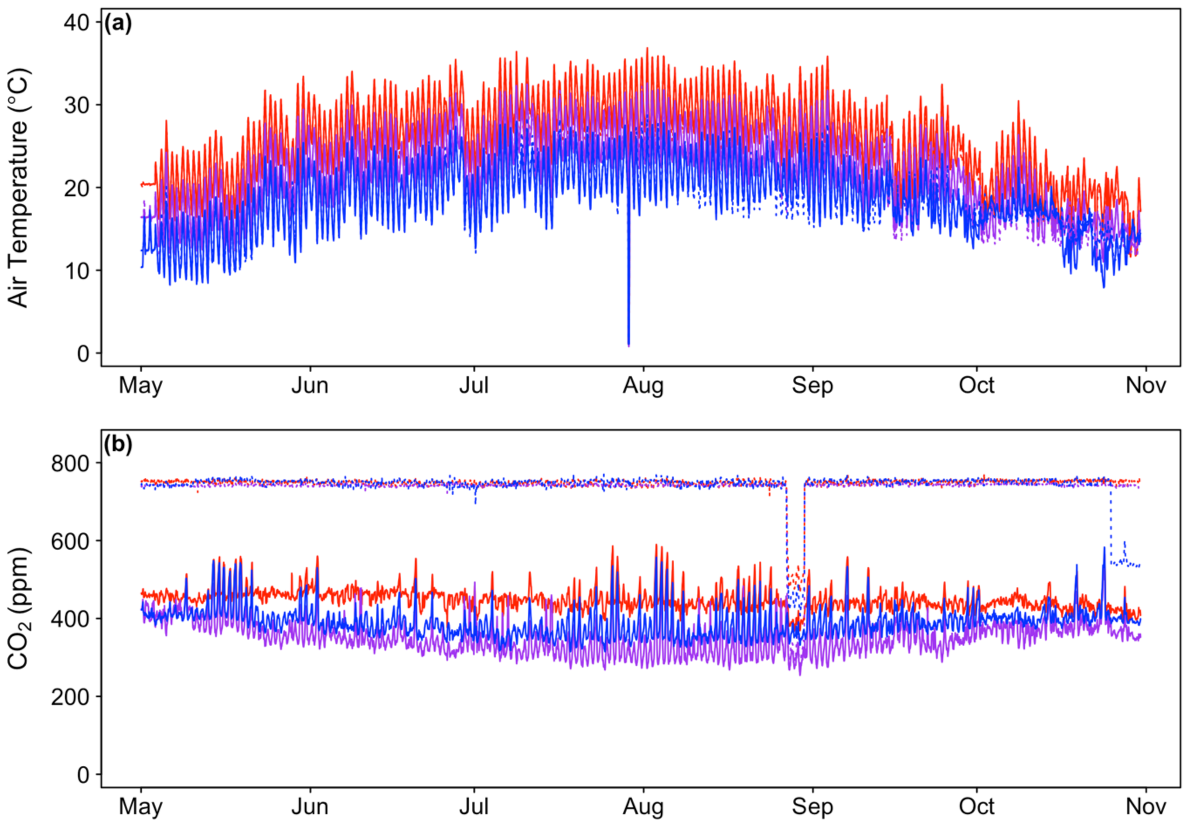

Supplemental Figure S1. Environmental treatments in each of the six glasshouses for the duration of experiment 1. (a) Air temperature, and (b) CO_2 concentrations. Colors represent the temperature treatments, with ambient temperature (T0) in blue, ambient +4°C (T4) in purple, and ambient +8°C (T8) in red. Solid and dashed lines represent the ambient CO_2 concentration treatments (AC) and elevated CO_2 concentration treatments (EC), respectively.

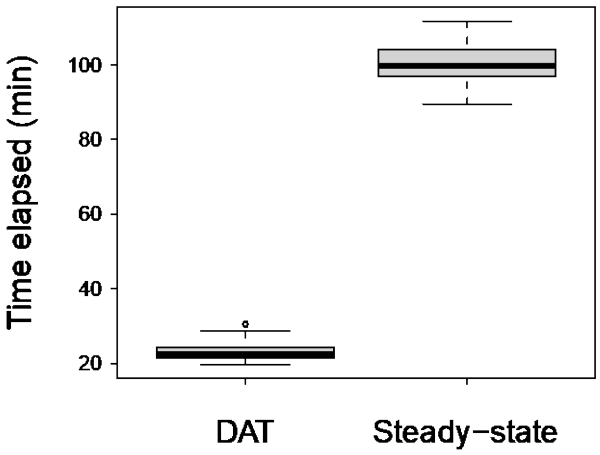

Supplemental Figure S2. Total time taken to perform the DAT compared to the steady-state Laisk method in paper birch (*Betula papyrifera*). The boxplots represent the median as well as the first and third quartiles. Whiskers delimit the range for each group, with outliers falling outside 1.5 x the interquartile range marked by points. n = 32 – 35.

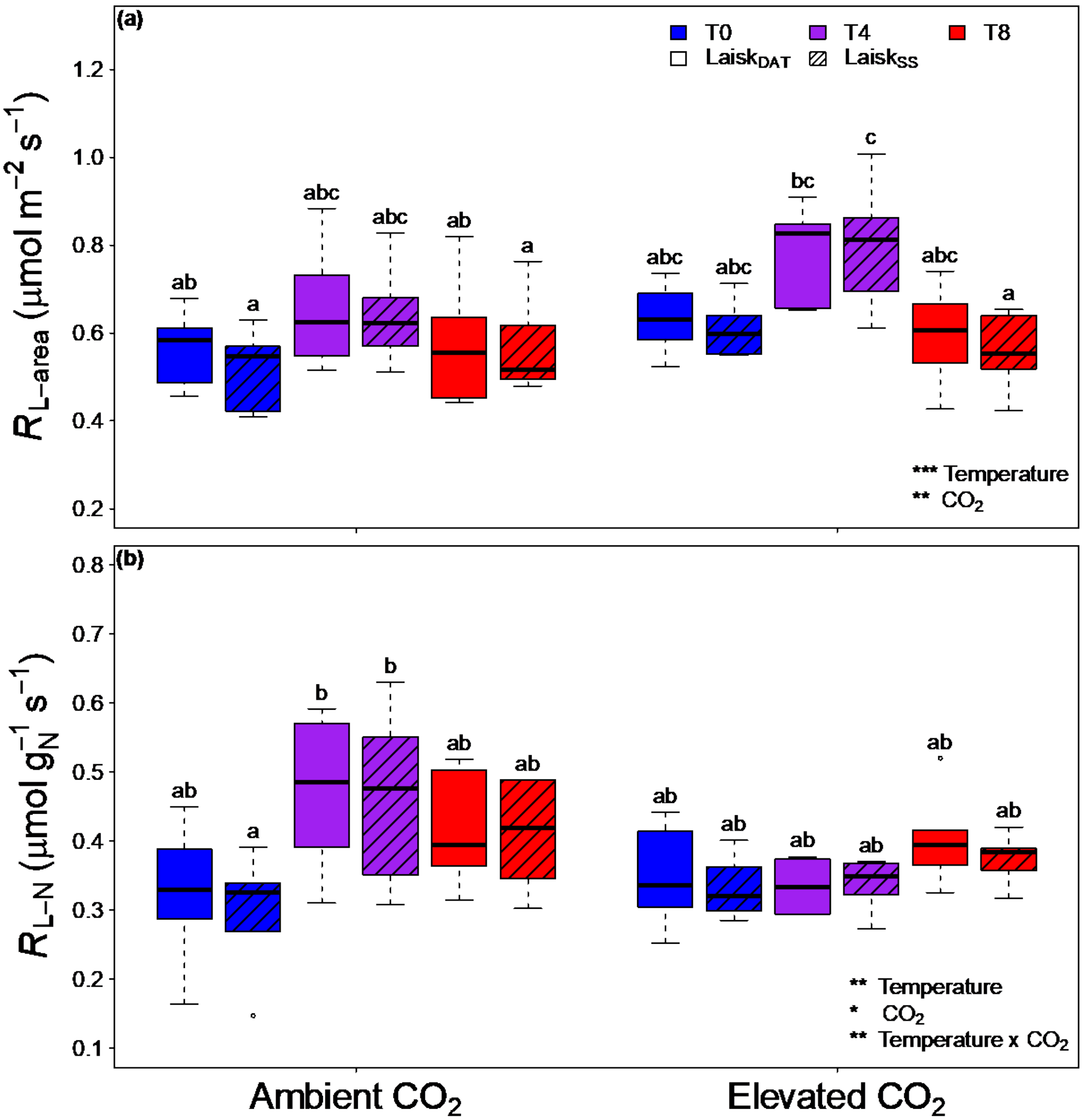

Supplemental Figure S3. Respiration in the light estimated according to the DAT and the steady-state Laisk methods (Laisk$_{\mathrm{DAT}}$ or Laisk$_{\mathrm{SS}}$; respectively) on paper birch (*Betula papyrifera*) from each of the six environmental treatments. (a) Area-based respiration in the light ($R_{\text{L-area}}$) and (b) per nitrogen-based respiration in the light ($R_{\text{L-N}}$). Colors represent the temperature treatments, with ambient temperature (T0) in blue, ambient +4°C (T4) in purple, and ambient +8°C (T8) in red. Hatching denotes measurement method (either hatched to represent Laisk$_{\mathrm{DAT}}$, or not, representing Laisk$_{\mathrm{SS}}$). The boxplots represent the median as well as the first and third quartiles. Whiskers delimit the range for each group, with outliers falling outside 1.5 x the interquartile range marked by points. Significance of the main effects for each trait as determined by a three-way ANOVA are noted (*** p<0.001; ** p<0.01). Full ANOVA results are found in Table S1. Different letters denote significant pairwise differences (p<0.05) as determined via a Tukey post-hoc comparison. n = 5 – 6.

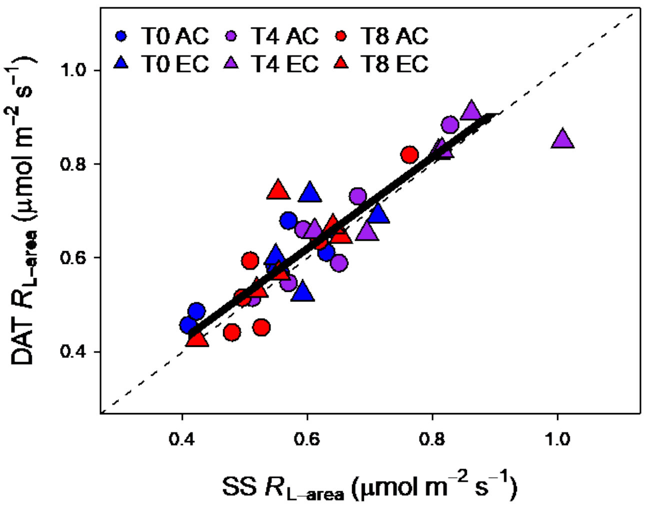

Supplemental Figure S4. 1:1 comparison of $R_{L\text{-}area}$ calculated according to the DAT and the steady-state (SS) Laisk methods on paper birch (*Betula papyrifera*). Colors represent the temperature treatments, with ambient temperature (T0) in blue, ambient +4°C (T4) in purple, and ambient +8°C (T8) in red. Circles denote the ambient CO_2 treatments (AC) and triangles the elevated CO_2 treatments (EC). The dashed line shows the 1:1 line. n = 5 – 6.

Supplemental Table S1. Summary of the three-way ANOVA in paper birch (*B. papyrifera*). *F*-values and *p*-values with temperature, CO_2 and measurement method as the main effects are shown. Traits analyzed were area- and per nitrogen-based respiration in the light ($R_{L\text{-}area}$ and $R_{L\text{-}N}$, respectively). Bold numbers represent $p<0.05$.

Effects	$R_{L\text{-}area}$		$R_{L\text{-}N}$	
	F-value	*p*-value	*F*-value	*p*-value
Temperature	13.477	**<0.001**	6.956	**0.002**
CO_2	8.964	**0.004**	5.357	**0.024**
Method	0.598	0.443	0.460	0.501
Temperature : CO_2	2.534	0.088	6.292	**0.003**
Temperature : Method	0.155	0.857	0.107	0.899
CO_2 : Method	0.026	0.873	0.000	0.985
Temperature : CO_2 : Method	0.125	0.883	0.095	0.910

Supplemental Table S2. Linear regression of the two methods, DAT and steady-state, used to estimate area-based respiration in the light ($R_{L\text{-}area}$) in paper birch (*Betula papyrifera*). A statistically similar slope (p>0.05) indicates no significant differences in the two methods.

Parameter	n	*p*-value	R^2	Slope	95% CI	H0 #1 Slope = 1	Intercept	95% CI	H0 #2 Intercept = 0
$R_{L\text{-}area}$	35	<0.001	0.784	0.972	[0.805, 1.172]	0.754	0.038	[-0.076, 0.151]	0.505

Number of samples (n), significance values (*p*-values), coefficients of determination (R^2), slope parameter estimates (Slope), 95% confidence intervals (95% CI), test of whether the slope equals 1 (H0 #1 Slope = 1), intercept parameter estimates (Intercept), and test of whether the intercept equals 0 (H0 #2 Intercept = 0).

Supplemental Text S1. Graphical methods for determining respiration in the light and the rubisco compensation point

The value for Γ^* (rubisco compensation point) that was modeled from the gas exchange data varied and pre-treating leaves with high or low CO_2 could create a difference of >0.7 Pa . In theory, Γ^* is that CO_2 concentration at rubisco at which the velocity of carboxylation is exactly ½ of the oxygenation velocity, assuming that one CO_2 is released for every two oxygenation events. The rubisco compensation point should be a function of rubisco kinetics and should not vary from one plant to another of the same species nor vary with growth conditions. We consider here two possible effects that could account for the variability of Γ^*.

1. Mesophyll conductance

It is not possible to measure Γ^* directly but instead C_i^* is measured, this is the CO_2 concentration in the air of the intercellular space of the leaf at which the velocity of carboxylation is exactly ½ of the oxygenation velocity. The difference from Γ^* is the drop in CO_2 caused by respiration in the light (R_L) and diffusion resistance of the cell, which is typically reported as the inverse, mesophyll conductance, g_m. The relationship between C_i^* and Γ^* is

$$C_i^* = \Gamma * - \frac{R_L}{g_m} \qquad \text{Equation 1}$$

Often, A, the CO_2 assimilation rate, is used instead of $-R_L$ but because rubisco is, in theory, at its compensation point $A = - R_L$. Thus, variations in observed C_i^* should be a function of R_L / g_m and the computed Γ^* should be constant for a given species. If R_L= 0.5 µmol m^{-2} s^{-1}, then g_m would cause C_i^* to vary as shown in Figure 1.

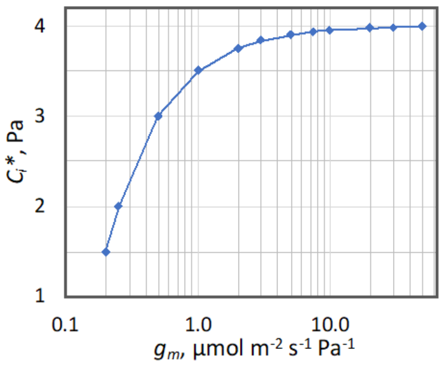

Figure 1. Variation in C_i* as affected by g_m. Assumptions include R_L = 0.5 µmol m^{-2} s^{-1}.

If g_m is greater than 1 µmol m^{-2} s^{-1} Pa^{-1}, it would be possible that errors in the determination of g_m could cause an error in Γ* determination of 0.5 Pa (C_i* value at infinite g_m - C_i* value at a g_m of 1 µmol m^{-2} s^{-1} Pa^{-1}). We consider it unlikely that estimates of g_m were inaccurate enough to account for the apparent variation in Γ*, especially because graphical methods for g_m determination become more robust at low values of g_m.

2. Glycine export from photorespiration

Carbon can leave the photorespiratory pathway. Under some conditions, this can lead to reverse sensitivity of photosynthesis to CO_2 and O_2 (Harley & Sharkey 1991). This has been modeled in detail by Busch *et al.* (2018)

$$A = \left(\frac{J}{(4 + (4 + 8\alpha_G + 4\alpha_S)\Phi_G)}\right)(1 - 0.5\Phi_G) - R_L \qquad \text{Equation 2}$$

where α_G is the amount of carbon that leaves photorespiratory metabolism as glycine and α_S is the amount that leaves as serine. Φ_G is $(1-\alpha_G)\cdot 2\cdot \Gamma^*/C$.

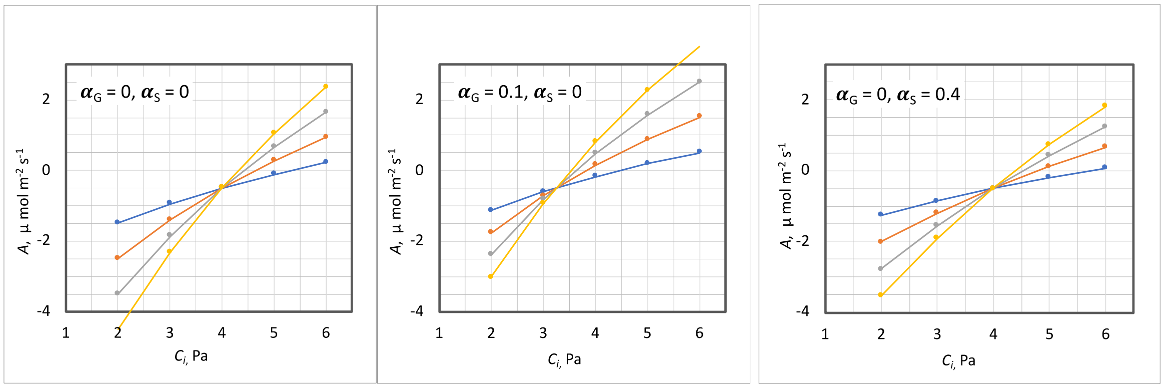

Figure 2. Modeled photosynthesis rates using Equation 2. R_L was 0.5 µmol m^{-2} s^{-1}, Γ^* was 4 Pa, and g_m was considered infinite, i.e. not used in this modeling.

With Φ_G and Φ_S equal to zero and g_m infinite, $C_i^* = \Gamma^*$ (Figure 2 left panel). With α_S equal to zero, α_G =0.1 and g_m infinite, C_i^* = 3.3 Pa, or 0.7 Pa below Γ^* (Figure 2 center panel). With α_S equal to 0.4, a value often found in fitting exercises, $\alpha_G = 0$, and g_m infinite, $C_i^* = \Gamma^*$ (Figure 2 right panel). The difference between the effect of α_G and α_S is because when glycine is exported, the CO_2-liberating step is bypassed but not when serine is exported. The effect of just 10% of the carbon in glycine leaving photorespiratory metabolism is similar to the effect on apparent Γ^* seen in response to pre-treating with either high or low CO_2.

In neither case was the estimation of R_L affected.

REFERENCES

Busch F.A., Sage R.F. & Farquhar G.D. (2018) Plants increase CO_2 uptake by assimilating nitrogen via the photorespiratory pathway. *Nature Plants* **4**, 46-54.

Harley P.C. & Sharkey T.D. (1991) An improved model of C_3 photosynthesis at high CO_2: Reversed O_2 sensitivity explained by lack of glycerate reentry into the chloroplast. *Photosynthesis Research* **27**, 169-178.
